# Digital technology supporting English learning among Indonesian university students

**DOI:** 10.1186/s41039-022-00198-8

**Published:** 2022-06-08

**Authors:** Didin Nuruddin Hidayat, Jee Young Lee, Jon Mason, Teguh Khaerudin

**Affiliations:** 1 Department of English Education, State Islamic University (UIN) Syarif Hidayatullah Jakarta, Jl. Ir. H. Juanda No. 95, Jakarta, Indonesia; 2 News and Media Research Centre, Faculty of Arts and Design, University of Canberra, 11 Kirinari St, Bruce, ACT 2617 Australia; 3 College Indigenous Future Education Arts & Society, IFEAS – Education, Charles Darwin University, Ellengowan Dr, Casuarina, NT 0810 Australia

**Keywords:** English learning, Online learning, COVID-19, Digital learning, Technology use

## Abstract

Worldwide, information technology now plays a substantial role in the daily life of most people, whether in the developed world or in developing countries, such as Indonesia. In this fourth most populated country in the world, information technology is widely used for everyday communications and entertainment purposes, as well as for supporting education. Using a survey of 496 students enrolled in a university in Jakarta, this paper reports on a study aimed at assessing the experiences of young Indonesian students undertaking online learning and the potential of this platform for English learning. The findings show that online activities, skills, and perceived usefulness were positively correlated with positive experiences of learning English online. In particular, the perceived usefulness of the Internet and the ability to use different functions of digital devices and applications had a stronger correlation with increased benefits of online English learning. The study generates implications for Indonesian education suggesting a review of the roles of English instructors in promoting English learning through technology, improvement in English instructors’ skills in utilizing technology in their teaching, and support of relevant stakeholders as well as the preparation of English teacher preparation programme to support pre-service teachers for teaching with technology.

## Introduction

Motivation for this study emerged over time, but principally because of the rapid uptake of digital technology in Indonesia in recent years. The research reported on in this paper commenced prior to the outbreak of COVID-19; however, the conditions created by this outbreak highlight the significance of several findings. Since its beginning in late 2019, the COVID-19 pandemic has spread throughout most of the world. Familiarly known as ‘Corona’ in Indonesia, COVID-19 has spread to 215 countries, claimed over 5 million lives, and around 254 million people have been infected (World Health Organization, 2021). The social order, which had stood strong for decades, has suddenly become vulnerable to economic collapse. Maintaining a viable education system has emerged as a priority focus for governments, and in Indonesia there has been a widespread effort to deliver emergency remote education using digital technology.

This corona pandemic can be seen as a catalyst for people to adapt quickly to unexpected changes, such as through social interaction, work, and learning. The Indonesian government has impacted these three domains through policies on social distancing, physical distancing programmes, and Large-Scale Social Restrictions (or PSBB) (Azhari & Fajri, 2021). Such policies are common worldwide (Nicola et al., 2020) and align with the argument that humans are the most adaptive living things on earth (Harari, 2014). Furthermore, humans have turned to technology, especially information technology and communication media, to help them navigate the unfamiliar territory of social life with restricted physical interaction.

While present in public discourse prior to the global financial crisis of 2007, the ‘New Normal’ is a prominent term emerging from the pandemic that summarizes the extent of social and economic challenges (Dziuban et al., 2018; El-Erian, 2010; Muhyiddin, 2020). Accordingly, new behaviours are being developed or becoming the New Normal. In educational contexts, even though remote teaching as the consequence of social distancing policy may take different forms (Arsendy et al., 2020; Carpenter & Dunn, 2020), many schools and teachers have turned to digital technologies for online remote teaching (Dhawan, 2020; Lie et al., 2020). Even teachers who were previously reluctant to use digital technologies in their teaching are now forced to shift to emergency online teaching and to adapt their pedagogy (Trust & Whalen, 2020). In other words, the global health crisis has paved the way for a new norm in schooling, that is the utilization of various digital applications and learning resources available via the Internet for teaching and learning.

Digital applications and resources are now even more accessible with the increasingly more affordable digital technology devices. Low-end smartphones and laptops are now affordable by many people from low- and middle-income households opening their access to the digital world (Bonfadelli, 2002). Many people in Indonesia own more than one digital device resulting in more than 300 million mobile connections as of January 2021 exceeding the country’s total population of 274.9 million (Kemp, 2021). This has put Indonesians as one of the leading adopters of digital technology products. For example, recent studies rank Indonesia highly on the lists of the number of social media users, i.e. the second largest users of Facebook, and the third of Twitter (Anwas et al., 2020; Ida et al., 2020; Utomo et al., 2013). Even though the mobile connections are still concentrated in Java Island (Eloksari, 2020), an island inhabited by more than half of Indonesia’s population, the data show that there is a big opportunity for the utilization of digital technologies to bring changes, such as changes in the ways Indonesians teach and learn English. It is also notable that the current Minister of Education in Indonesia was previously a successful entrepreneur using social media as a platform.

Many Indonesian English teachers and learners, especially those living in big cities in Java Island, are, indeed, already familiar with using different forms of digital technologies for their English language teaching and learning. For example, Indonesian English teachers have used the Internet to access information and resources for teaching (Cahyani & Cahyono, 2012), social media for improving students’ writing (Rodliyah, 2016) and LMS applications for promoting language learner autonomy (Ardi, 2017; Pasaribu, 2020). From his observation of English language learners in rural Indonesia, Lamb (2013) reported how young Indonesian students used their mobile phones to access the Internet and social media, and how they learn English from doing it. For many Indonesian youths, access to digital technologies enables them to be more independent in their learning and facilitate the development of a global outlook. Over time, as a consequence of these rapidly changing trends, it is no doubt that the digital industries will increasingly target younger generations as their potential market. Moreover, it is well known that digital technologies have been instrumental in fostering self-directed learning and new pedagogical approaches such as the ‘flipped classroom’ and applications of interest-driven creator theory (Bullock, 2013; Chan et al., 2018; Karakas & Manisaligil, 2012).

### Literature review

With the rapidly growing access to the Internet comes a considerable expectation that learners of English as a Foreign Language (EFL) take the maximum advantages from it for their learning agenda. Learning English in an EFL classroom context is often constrained by time restrictions, predefined topics, limited learning materials and scarce opportunity for practicing the language. The advancement of information and communication technology (ICT) and the ease of access to the Internet has delivered solutions to those problems and is poised to deliver more through artificial intelligence (Ismail & Hastings, 2021). The Internet, for example, has provided learners with opportunities to learn English from authentic materials and to experience the authentic use of English when conversing with other learners or native speakers (Marull & Kumar, 2020). Furthermore, the synchronous and asynchronous communication technologies of the Internet allow language learners to use English for online social collaboration, discussion, and intercultural give-and-take (Dooly, 2017, as cited in Marull & Kumar, 2020). These are very favourable in language education because of the mix of informal interaction and reflective writing opportunities.

English learning materials on the Internet are currently even more accessible with the extensive use of digital devices that allow ubiquitous language learning. Recent studies on online language learning have seen a rise in popularity in using mobile devices for language learning, shifting away from the trend from online language learning using computers (computer-assisted language learning/CALL) (Andujar et al., 2020; Şad et al., 2020; Shih & Chang, 2020). Indeed, according to a meta-analysis review of peer-reviewed journal articles and dissertations by Sung et al. (2015), learning languages with mobile devices is more effective than learning with computers or laptops in terms of cognitive and affective learning outcomes. Some advantages of using mobile devices for language learning come from their portable nature, increasing number of apps for learning, and their accommodation for different learning styles and preferences (Kang & Lin, 2019). Their portability makes mobile devices suitable for learning both in and beyond the language classroom.

### Interest and satisfaction with online learning

Having access to the Internet alone, however, does not guarantee the effective use of technology for online learning. For optimized learning, access to online services should be accompanied by appropriate attitudes and behaviours of learners. Chan et al. (2018) argue that ‘interest’ also plays an essential role in effective learning and is a key to transforming the examination-driven systems of education pervasive throughout Asia. Online learning can be very challenging for learners because they need to be able to study on their own without any physical supervision they would otherwise have in a traditional brick and mortar classroom setting. To succeed, learners need to possess a strong motivation to learn independently. Motivation, like in any other mode of learning, plays an essential role in effective online English learning. Student interests, as one of the motivational variables, help students curb difficulties in learning (Deci & Ryan, 2004), enhance supports from learning system and materials (Rotgans & Schmidt, 2014), and help students make sense of the learning tasks (Hidi & Renninger, 2006).

Likewise, learning satisfaction has often been regarded as one of the critical indicators of a successful online learning programme. According to Horzum (2015), ‘learners who are satisfied with an online learning program would feel fulfilled and good about different features of supports they received from the program’ (*p*. 506). In this sense, satisfaction is a desirable psychological response that is directly influenced by both the human and design dimensions of an online learning programme (Eom, 2019). Previous studies on the effectiveness of online learning programmes often include the learners’ satisfaction as one of the dependent variables (Eom, 2019; Goh et al., 2017; Guest et al., 2018; Horzum, 2015). In this study, therefore, the learners’ interest and satisfaction when experiencing an online English learning programme are the desirable dependent variables.

### Engagement and digital media literacy

A successful online learning session also requires that learners display positive learning behaviours. Jewell (2006) maintained that the freedom to access online learning resources ubiquitously could facilitate effective language learning only if the students keep engaged with the online resources and activities and if they take more responsibility for their learning. In this view, the degree to which students engage with and manage their online activities are important factors to their success. Learning engagement is evident when students make efforts, mentally and emotionally, to complete learning tasks at hand (Schunk & Mullen, 2012). That learning engagement contributes to successful learning is confirmed by many studies (Buelow et al., 2018; Christenson et al., 2012; Halverson & Graham, 2019) which led Sinatra, Heddy, and Lombardi (2015, as cited in Halverson & Graham, 2019) to call learning engagement as ‘the holy grail of learning’. In an online learning environment, where students need to take more initiative, learning engagement is considered an even more important factor in learning success.

Completing learning tasks provided through online media bears the consequence that the students must know how to navigate the digital devices and contents. This digital media literacy is an essential requirement for maximum utilization of technology (Park & Burford, 2013) and effectiveness in an online learning environment (Tang & Chaw, 2016). Park (2011, as cited in Park & Burford, 2013) defines digital media literacy as ‘the necessary skillsets required to thrive in the digital media environment’. It entails not only the ability to use digital devices but also the ability to manage, modify, and evaluate information retrieved through the devices as well as the capacity to behave appropriately in an online environment (Park & Burford, 2013; Tang & Chaw, 2016). The European Framework for Digital Literacy defines digital literacy as:

‘… the awareness, attitude and ability of individuals to appropriately use digital tools and facilities to identify, access, manage, integrate, evaluate, analyse and synthesize digital resources, construct new knowledge, create media expressions, and communicate with others, in the context of specific life situations, in order to enable constructive social action; and to reflect upon this process.’ (Martin, 2006 as cited in Tang & Chaw, 2016, p. 56)

Park and Burford (2013) theorize three dimensions of digital media literacy, namely *access*, *understanding*, and *create*. *Access* refers to the knowledge of the capability of digital media and the ability to operate digital devices. *Understanding* refers to the ability to decipher mediated messages, including the ability to read the motives of the message production and to identify fact from fiction. Lastly, the *creative* dimension of digital media literacy entails the ability to search, share, synthesize and create information. This digital media literacy is an essential requirement for maximum utilization of technology (Park & Burford, 2013) and effectiveness in an online learning environment (Tang & Chaw, 2016b). Considering the ubiquitous nature of innovation with digital technologies, however, meanings associated terminology such as ‘digital literacy’ will likely evolve. Thus, Mirra et al. (2018) also highlight that engaging with the digital environment is no longer just about consuming digital services but participating in producing artefacts with students becoming ‘digital civic agents’.

### Assisting teachers

For teachers, making use of digital technology during instruction also brings challenges. Not only must they develop their own skills in usage but they must also integrate such skills into their teaching. Scholars have theorized different models of digital capabilities required for teachers to effectively use of new technologies in teaching. Two frameworks most frequently used to assist teachers in making effective use of digital capabilities in teaching contexts are TPACK and SAMR (Falloon, 2020). Mishra and Koehler (2006) theorize TPACK (technological pedagogical content knowledge) suggesting a multidimensional process of technology integration in teaching that requires teachers’ understanding of three types of base knowledge, namely pedagogical knowledge (PK), content knowledge (CK), and technological knowledge (TK), and the dynamic interactions between and among them within a certain context (Koehler et al., 2013; Mishra & Koehler, 2006). Alternatively, the SAMR (Substitution Augmentation Modification Redefinition) model by Puentedura (2006) suggests a more graduated approach to technology integration in teaching. While such frameworks are not explicit about what constitutes digital media literacy they are designed for teachers to appreciate the pivotal role that digital technology now plays in facilitating learning outcomes.

In many contexts of EFL learning, such as in Indonesia, the adoption of digital technology is pervasive. It has been, therefore, deemed essential to understand how digital technology has been utilized by the learners and how it has facilitated achievement of learning outcomes. Thus, the present study also aimed to identify the learning and development opportunities available through the Internet, specifically for English learning, by exploring how Indonesian young people *experience* online English learning and what other factors, including online engagement, digital literacy and perceived usefulness of the Internet, are associated with outcomes. Following many previous studies on online learning, the present study did not use summative assessment of the learning outcomes, such as course grades. Instead, learning outcomes used here refer to the learners’ increased interest in online learning and their satisfaction with the learning arrangement.

## Research design

This research was designed as an exploratory study in order to examine the roles of learning engagement and digital literacy in learning, in particular, online English learning settings. At the outset, it is recognized that such a study must consider the context of rapid innovation with digital technologies and communications. Thus, we adopted the concepts of learning engagement (Buelow, Barry, & Rich, 2018; Christenson, Reschly, & Wylie, 2012; Halverson & Graham, 2019) and digital media literacy (Park & Burford, 2013) to examine how these factors will play in online learning in an evolving digital environment.

- The following research questions were formulated:
- RQ1. Are online activities and skills related to experiences of learning English online?
- RQ1-1. Are the levels of online activities and digital literacy related to satisfaction with online English learning?
- RQ1-2. Are the levels of online activities and digital literacy related to interest in online English learning?
- RQ2. Is the perceived usefulness of the Internet related to experiences of online English learning?
- RQ2-1. Is the perceived usefulness related to satisfaction with online English learning?
- RQ2-2. Is the perceived usefulness related to interest in online English learning?

The study also aimed to shed light on developing understanding of contemporary use of the Internet by young people for English learning. It was expected that the results of this study might provide further strategic insight on how practitioners can integrate available online learning resources into the classroom.

## Method

This study used a self-administered questionnaire method as data collection. A paper-based survey was conducted in a public university in Jakarta, Indonesia between September and October 2016. Two trained graduate students recruited undergraduate students on the campus and then distributed and gathered the survey, answering questions if required. Ethical approval was granted by the University of Canberra before the data collection was conducted.

The study recruited participants using a convenience sampling method. The sample was taken from a university where the researchers had access to the resources including participants, research assistants and other administration supports. We selected this method due to its efficiency in conducting a survey study in terms of cost, time and other resources. A convenience sampling method is particularly useful to test out ideas about a subject during the exploratory stages of a research project (Blumberg et al., 2014). This study was designed as an exploratory project, examining the roles of digital media literacy and online engagement in learning English online among university students; therefore, we selected the sampling method as an efficient approach considering the time and cost given.

Participant consent was acquired prior to completing a questionnaire. The participants were informed that their participation was voluntary and confidential, and that they had the right to withdraw from the study at any time without any adverse effect. Data collection was managed within the estimated time frame as to minimize the potential of boredom and fatigue. To achieve this objective, pilot-tested were carried out to measure the estimated time for completing the questionnaire.

### Measurements

The participants’ experiences of learning English online are measured in terms of their satisfaction with and interest in the mode of learning. Referring to Elliott and Healy (2008), learning satisfaction is defined as ‘a short-term attitude that results from an evaluation of a student’s educational experience and results when the actual performance meets or exceeds the learner expectations’ (p.2). In this study, we adopted Lin’s (2008) and Song and Zinkan’s (2008) measurement of learning satisfaction. We measured the levels of satisfaction with online English learning with four items on a 5-point Likert-type scale based on the following: strongly disagree (1), somewhat disagree (2), neutral (3), somewhat agree (4), and strongly agree (5) (*α* = 0.866).

In the meantime, the participants’ interest in learning English online is measured with a questionnaire adapted from the interest in learning with social media measurement from Hong et al. (2016). The measurement was based on Hidi and Renninger’s (2006) situational interest, i.e. ‘focused attention and the affective reaction that is triggered in the moment by environmental stimuli, which may or may not last over time’ (p. 113). Four items were measured on a 5-point Likert-type scale: strongly disagree (1), somewhat disagree (2), neutral (3), somewhat agree (4), and strongly agree (5) (*α* = 0.830).

**Online activities** Participants’ different engagements with online activities were measured with 14 items designed to identify how often the participant engages in online activities. In this study, three different types of online activities are identified, namely activities related to searching for information or learning, entertainment, and communication. When responding to the previously stated items, the participants were requested to ponder common devices they use, for instance, computers, tablets, and smartphones. Answers were reported on an eight-point Likert-type scale ranging from (1) ‘never’, (2) ‘less than every few months’, (3) ‘every few months’, (4) ‘every few weeks’, (5) ‘1–2 days a week’, (6) ‘3–5 days a week’, (7) ‘about once a day’, to (8) ‘several times a day’.

Both device literacy and content literacy, adapted from Park and Burford (2013)**,** were employed to measure digital media literacy. The measurement includes the participants’ understanding of the device, understanding of the contents, and the ability to create content. There are three (3) items for each dimension on a 5-point Likert-type scale: not at all (1), not much (2), somewhat (3), quite a bit (4), and very well (5).

**The perceived usefulness of the Internet** The perceived usefulness of the Internet based on Yang and Yoo's (2004) study was measured. Here, adapting Yang and Yoo’s (2004) definition, perceived usefulness refers to the degree to which students believe that using the Internet would enhance their learning. Five items were measured on a 5-point Likert-type scale based on the following: strongly disagree (1), somewhat disagree (2), neutral (3), somewhat agree (4), and strongly agree (5) (*α* = 0.869).

**Demographics.** Gender and age variables were measured.

## Data analysis

Using SPSS 18.0 software, descriptive statistics and frequency analysis were performed to identify the basic statistics of demographics. We used Spearman’s correlation analysis in order to examine the associations between variables identified.

### Participants

In total, data from 524 respondents were collected, of which 496 respondents were finally used for data analysis after data checking. Of the final respondents, 66.3% (326) were female and 33.7% (166) were male. Respondents aged 16–17 were 9.1% (45), 18, 36.6% (181), 19, 29.1% (144), 20, 16.8% (83) and 21 or over, 8.3% (41). The distribution of parents’ monthly income (IDR) among respondents is as follows: ‘less than 1.000.000–2.500.000’, 29.1% (143), ‘2.500.001–4.500.000’, 36.5% (179), ‘4.500.001–11.000.000′ 25.9% (127), and ‘more than 11.000.000’, 8.6% (42). 31.9% (158) were a freshman, 41.4% (205) were a sophomore, 18.2% (90) were a junior, and 7.0% (34) were a senior.

## Results

Spearman’s correlation analysis was conducted to examine the relationship between online English learning experience and online learning activities, digital skills, and perceived usefulness of the Internet. In general, the correlation test showed that online activities, skills, and perceived usefulness were all positively correlated with experiences of online English learning.

### Online activities and experiences of learning English online

To see if there was a relationship between the frequency of doing online activities and experiences of online learning, a correlation analysis was conducted between the students’ frequency of doing three different online activities, namely information/ learning, entertainment, and communication, and two variables of online English learning experiences (satisfaction and interest). Overall, the students’ engagement with different activities on the Internet positively correlated with satisfaction with and interest in online English learning (Table 1). In particular, frequent online activities for communicating with others had the higher correlation with increased satisfaction with (*r* = 0.187, *p* < 0.01), and frequent online activities for information searching/learning had the higher correlation with increased interest in learning English online (*r* = 0.185, *p* < 0.01). Among the three activities, going online for entertainment had the lowest correlation with interest in online English learning (*r* = 0.135, *p* < 0.05). The finding shows that students who engage more often with communication-related online activities are more likely to have positive experiences of learning English online.

**Table 1:** Relationship between online activities, skills and perceived usefulness, and experiences of online English learning

		Interest	Information/ learning	Entertainment	Communication	Device understanding	Content understanding	Content Create	Device Create	Usefulness
Experience	Satisfaction	0.707**	0.141*	0.142*	0.187**	0.346**	0.349**	0.159**	0.288**	0.513**
	Interest		0.185**	0.135*	0.172**	0.353**	0.321**	0.117*	0.223**	0.463**
Online activity	Information/learning			0.261**	0.484**	0.271**	0.278**	0.055	0.153**	0.257**
	Entertainment				0.306**	0.188**	0.263**	0.121**	0.199**	0.147**
	Communication					0.305**	0.278**	0.193**	0.261**	0.221**
Digital literacy	Device understanding						0.59**	0.302**	0.421**	0.408**
	Content understanding							0.35**	0.441**	0.338**
	Content Create								0.508**	0.132**
	Device Create									0.302**

** *p* < .01, * p < .05

### Digital media literacy and experiences of learning English online

In this study, digital media literacy is divided into three dimensions, i.e. device understanding, content understanding, and content creation. Generally, digital media literacy was positively associated with online learning experience. As shown in Table 1, the ability to navigate digital devices (satisfaction, *r* = 0.346, *p* < *0.0*1, interest *r* = 0.353*, p* < 0.01) and to understand digital contents (satisfaction, *r* = 0.349, *p* < 0.01, interest, *r* = 0.321, *p* < 0.01) had higher correlation with their online English learning experiences than other digital skills such as digital content and device creation.

### Perceived usefulness and experiences of learning English online

The correlation test shows that learners’ belief in the benefits of the digital technology for English learning online is strongly correlated with the degree of their interest and satisfaction with the online learning programme (satisfaction *r* = 0.513, *p* < 0.01, interest *r* = 0.463, *p* < 0.01). The correlation coefficient between online learning experiences and perceived usefulness is, indeed, higher than between experiences and both different online activities and digital media literacy. It suggests that those who perceive the Internet as a useful tool and experience ease of Internet use are more likely to have improved satisfaction and interest in online English learning.

## Discussion

The present study investigated how experiences of online English learning relate to different online activities, digital skills, and perceived usefulness of the Internet for learning. It specifically examined whether different online activities, different types of digital skills, and perceived usefulness of the Internet increased satisfaction of and interest to online English learning. The results show perceived usefulness of the Internet strongly predicts increased benefits of online English learning in terms of both interest and satisfaction. This result is in line with previous studies (Wang et al., 2016). Learning interest describes how learners respond to a particular topic leading to learners' attention (Hong et al., 2016), and learners who have a higher interest in learning are more likely to possess positive attitudes towards learning results and display high satisfaction (Zhang et al., 2006)**.**

Findings clearly show perceived usefulness and ease of use of the Internet increase interest in online English learning, implying the significance of attitudes towards the Internet by young Indonesians when it comes to learning online. Furthermore, our findings suggest that those who are confident in understanding digital devices, such as PCs and smartphones, are more likely to experience positive outcomes associated with English learning online. It is worth noting that device literacy is one of the strongest predictors of online learning satisfaction overall (Lee & Hidayat, 2019).

As younger generations positively view the Internet as a significantly valuable bridge to their English learning, it may indicate that the role of the English teachers, lecturers, or other instructors needs to be revisited. While the performing English instructors are always seen as the primary source of knowledge whom students should follow, a huge number of valuable English learning sources available on the Internet could now be considered as the gate for younger generations to have deep self-exploration to any English learning materials they are keen to learn. To provide an example, English instructors could perform as a content facilitator, advisor, co-learner, assessor, or resource provider (Bjekić et al., 2014; Denis et al., 2004; Nguyen, 2019), maximizing student’s opportunity to be independent learners who could identify what to learn, why they learn it, and how they best learn it (Benson, 2011). In this matter, English instructors need to adjust their role to serve as guides in students’ journeys. While students are encouraged to become autonomous learners, teachers’ roles as expert learners become crucial in guiding their students on how to learn online, both inside and outside the classroom (Benson, 2011) (Grow, 1991). Therefore, if students lack an understanding of why and how to effectively self-direct their own learning, it might be challenging to maximize their potential (Little, 1995). This becomes ever more challenging in the contemporary digital environment because new innovations bring new affordances that are not necessarily easily identified or appreciated by educators and learners without giving sufficient time to explore what is possible.

Drawing from the above-mentioned findings, the present study highlights some major implications. Firstly, since younger generations are generally confident with their digital skills and may find it less problematic when they are necessarily required to adapt to technology, language instructors need to adjust to their students’ level of digital skills. For example, finding which digital tools might be enthusiastically used in their teaching and learning.

Rapid penetration of digital technology into classrooms is expected to revolutionize the ways we educate students. Concerning English language learning, the expectation to make the most use of digital technology is growing in parallel with the increased demand for improved digital skills and competencies (Evans, 2009; Malinina, 2015). The demand is true for both the students and the teachers. For teachers, the adoption of technology in the classrooms suggests that language instructors re-examine their role in teaching and learning activities. Language instructors are required to explore various teaching styles and media for learning, which are more engaging to their students. Often, however, teachers’ use of new technology does not lead to significant changes in their teaching delivery. As pointed out by Howard & Mozejko (2015), teachers’ use of interactive whiteboards can just resemble the teaching delivery mediated by the same old whiteboard. Selwyn (2011) maintains that adopting new educational technologies should mean that teachers shift their role ‘from matters of “teaching” to matters of coordinating and designing processes of “delivery”’ (p. 124).

As English instructors are required to adapt to the changes in teaching with the advancement of digital technology, it is no doubt that substantial support for the English teachers, lecturers, and other instructors should also be provided by relevant stakeholders. Instructors with improved digital capabilities will likely have higher confidence in using new technologies and facilitate positive student learning outcomes (Koh et al., 2016). When instructors demonstrate confidence using digital technologies learning can be experienced as more interesting and relevant to different learner needs (Hossain et al., 2020). Therefore, given the rapid development of the digital environment, encouraging both pre-service and practising teachers to make use of frameworks such as TPACK and SAMR should assist their efforts at effective integration of technology into their teaching.

While participants of this study agreed on the benefits of technology for their English learning, some studies (Maulida & Lo, 2013; Silviyanti & Yusuf, 2015) provided contradicting findings to the technology readiness in some regions in Indonesia, for example in Banda Aceh. Maulida and Lo (2013) argued that issues such as financial hardship, limited ‘competent’ human resources, and lack of government support remained the challenges that schools encounter in adopting the technology. Regular training inviting prominent practitioners, for example, could be taken as continuous professional development useful to maintain English instructors to be updated with current changes so that teachers and students could be maximizing new technology products for the benefits of their English classroom. In contrast, experiencing recent technology, they could learn English as well.

Lastly, as the growing attention increased, this study urges that English teacher education programmes prepare prospective teachers to be ready for future challenges related to technology in language teaching. That student teachers may find it efficient to learn English from available online sources should also be supported by providing examples of how technology could be maximized for their future English classroom. English teacher educators should model how digital skills are implemented in language classrooms. For example, English teacher educators could address how to teach specific learning materials by using several technology devices. The student teachers’ first-hand experiences in using the technology for their own learning may, in turn, determine the success of technology integration in their future teaching.

## Conclusion

The present study has discussed the perceived usefulness of the Internet for English learning and the importance of digital literacy and skills in Indonesia. While it was initiated prior to the COVID-19 pandemic, both its focus and findings align with other studies around the world. Thus, it was found that both perceived usefulness and digital literacy contribute to the participants’ level of confidence in using digital technology for their English learning—this can also be regarded as a virtuous cycle involving interest in and familiarity with the digital environment. Findings suggest that learners’ positive perception and confidence in online learning should be capitalized by EFL teachers for further integrating online technologies for language learning. It is deemed of utmost importance that teachers revisit their roles in the technology-integrated learning context, making use of frameworks such as TPACK and SAMR to be better informed in ways of technology integration. A learners’ ability to access information through the Internet shifts the teachers’ focus from proving information to coordinating and designing the learning processes. This also implies that teachers are required to develop their capacity for effectively using new technologies in their teaching. Effective teaching with technology requires that teachers develop their digital capabilities. Lastly, findings also highlight the need for supports from relevant stakeholders, and the readiness of EFL pre-service teacher programmes to train their students for future real teaching contexts.

While the findings in this study are informative and encouraging, the research method does have a limitation. This study was conducted in a university in Indonesia using a convenience sampling method. Therefore, the results may not adequately represent Indonesian young people more broadly, and generalizations from the results of this study should be made cautiously. It is also expected that future work could address in-depth exploration to seek further information from the perspectives of the English teachers, lecturers, or other instructors to gain a more comprehensive picture of the phenomenon.

## Data Availability

Data can be obtained from the corresponding author.
